# Programmed cell death ligand 1 (PD-L1) blockade attenuates metastatic colon cancer growth in cAMP-response element-binding protein (CREB)-binding protein (CBP)/β-catenin inhibitor-treated livers

**DOI:** 10.18632/oncotarget.26892

**Published:** 2019-04-30

**Authors:** Yosuke Osawa, Ekumi Kojika, Koji Nishikawa, Masamichi Kimura, Shigenori Osakaya, Hiromi Miyauchi, Tatsuya Kanto, Yutaka Kawakami, Kiminori Kimura

**Affiliations:** ^1^Department of Hepatology, Tokyo Metropolitan Cancer and Infectious Diseases Center Komagome Hospital, Bunkyo-ku, Tokyo 113-8677, Japan; ^2^The Research Center for Hepatitis and Immunology, National Center for Global Health and Medicine, Ichikawa 272-8516, Japan; ^3^Division of Cellular Signaling, Institute for Advanced Medical Research, Keio University School of Medicine, Tokyo 160-8582, Japan

**Keywords:** PD-L1, β-catenin, CBP, colon cancer, metastatic liver cancer

## Abstract

Immune checkpoint blockade with specific antibodies can accelerate anti-tumor immunity, resulting in clinical responses in patients with various types of cancer. However, these antibodies achieve only partial tumor regression. Thus, a wide variety of treatment combinations based on programmed death-ligand 1 (PD-L1) pathway inhibition are under development to enhance such therapeutic effects. In this study, the effects of combination treatment using PRI-724, a selective inhibitor of CBP/β-catenin, and an anti-PD-L1 antibody were examined in a mouse model of colon cancer liver metastasis. Mice were inoculated with SL4 colon cancer cells to produce metastatic liver tumors. The combination treatment resulted in regression of tumor growth, whereas monotherapy with each treatment individually failed to exhibit any anti-tumor activity. In addition, co-administration of the inhibitor and antibody induced CD8^+^CD44^low^CD62L^low^ cells and interferon (IFN)-γ production in CD8^+^ T-cells in the liver compared with that in control mice. Administration of an anti-CD8 antibody mitigated the anti-tumor effects of the combined treatment of PRI-724 and anti-PD-L1 antibody. In conclusion, targeting CBP/β-catenin, combined with PD-1/PD-L1 immune checkpoint blockade, shows potential as a new therapeutic strategy for treating liver metastasis during colon cancer.

## INTRODUCTION

Immune checkpoint blockade has been reported to have anti-cancer effects with antibodies (Abs) against programmed cell death 1 (PD-1) and programmed death-ligand 1 (PD-L1) being effective in types of several cancer [1–3]. The expression level of PD-1 ligands is increased in various cancers, resulting in cancer cell resistance to elimination by tumor-specific T cells [1]. The anti-tumor activity of an anti-PD-1 Ab has been observed in non-small cell lung cancer, melanoma, and renal cell cancer; however, no response was observed in colorectal cancer [2]. Similarly, the anti-tumor activity of an anti-PD-L1 Ab was observed in melanoma, non-small cell lung cancer, renal cell cancer, and ovarian cancer, but no response was observed in colorectal cancer [4]. However, in most cases, anti-PD-1/PD-L1 monotherapies result in only partial tumor regression [1]. Therefore, combination therapies based on PD-1/PD-L1 blockade may be required to increase the therapeutic effects of these agents. In melanoma, tumor regression based on the anti-tumor effects of an anti-PD-1 Ab requires the presence of CD8^+^ T-cells in the tumor [5] and activation of the β-catenin pathway in the tumor is inversely correlated with the extent of CD8^+^ T-cell infiltration [6]. Moreover, activation of β-catenin induces the expression of the transcriptional repressor AMP-dependent transcription factor-3 (ATF3), which suppresses the production of C-C motif chemokine ligand-4 (CCL4), leading to T-cell exclusion [6].

Wnt/β-catenin signaling is involved in virtually every aspect of embryonic development, as well as homeostatic self-renewal in adult tissues and is also associated with the pathogenesis of many human diseases such as colorectal cancer [7] and liver fibrosis [8, 9]. Moreover, immunosuppressive roles of activated β-catenin signaling have been reported in melanoma cells [10]. Following activation by upstream signaling from Wnt, β-catenin translocates to the nucleus. Nuclear β-catenin then recruits the Kat3 transcriptional co-activators cAMP-response element-binding protein (CREB)-binding protein (CBP) or EP300. Accordingly, the CBP/β-catenin antagonist ICG-001 has been shown to reduce the growth of cholangiocarcinoma in an animal model [11]. In addition, ICG-001 was found to increase sensitivity to cisplatin in platinum-resistant ovarian cancer cells [12]. PRI-724 is a second-generation specific CBP/β-catenin antagonist developed by Prism Pharma and is known to increase the expression of several chemokines in the liver during the resolution of liver fibrosis [8]. Colon cancer, one of the most common malignancies, frequently metastasizes to the liver and is often resistant to anti-PD-1 or anti-PD-L1 Abs [2, 4]. Therefore, in effort to increase the anti-tumor efficacy of PD-1/PD-L1 immune checkpoint blockade, we investigated the *in vivo* effects of PRI-724 treatment in combination with an anti-PD-L1 Ab on the progression of liver metastasis from colon cancer using an mouse model.

## RESULTS

### An anti-PD-L1 Ab combined with a CBP/β-catenin inhibitor decreased metastatic tumor growth in liver

**Figure 1:**
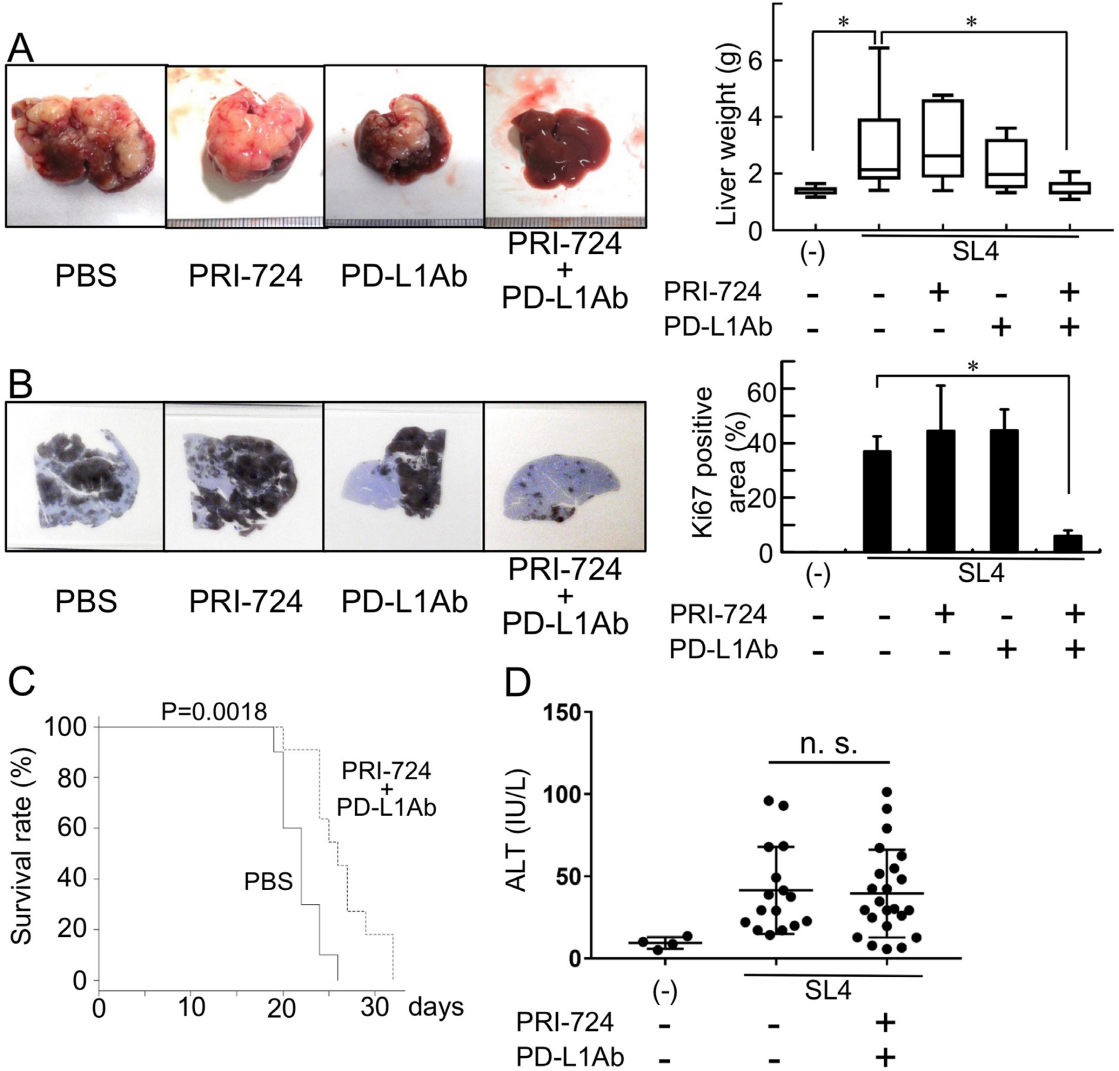
Anti-PD-L1 antibody (Ab) with a CBP/β-catenin inhibitor decreases metastatic tumor growth in the liver. Male C57BL/6J mice were intrasplenically injected with SL4 cells (5 × 10^5^ cells) and treated with or without anti-PD-L1 Ab and/or PRI-724. The animals were humanely sacrificed 14 days post inoculation (**A, B, D**). (A) The livers were excised and photographed (left panels). Liver weights were measured (right panel). (B) Expression of Ki67 in the metastatic liver tumor (loupe magnification) was examined by immunohistochemistry using an anti-Ki67 Ab. Intrahepatic tumor load is presented as Ki67-positive areas based on the measurement of two non-sequential for each animal (graph on right panel). The pictures shown are representative of at least four independent experiments. Results are provided as box-and-whisker plot or as means ± SD of data collected from at least four independent experiments. ^*^P < 0.05 based on the Kruskal-Wallis test (A) and one-way ANOVA test (B). (**C**) The survival rates of the animals are shown. Statistically significant differences were determined by performing a log-rank test. (D) Serum ALT levels were determined and the results are provided as means ± SD. n. s.; not significant.

To determine if the mouse colon cancer cell line SL4 expressed PD-L1, we performed immunohistochemical analysis and FACS using an anti-PD-L1 Ab. As shown in Supplementary Figure 1, the SL-4 cells did express PD-L1. To examine the anti-tumor effect of PD-1/PD-L1 immune checkpoint blockade on metastasis liver tumors, liver lesions were induced by the intrasplenic injection of SL4 cells and then PRI-724 (0.4 mg/mouse) and/or anti-PD-L1 Ab (200 μg/mouse) were administrated to these animals. Two weeks post inoculation, the liver weight and Ki67-positive tumor area were found to be increased in the PBS-treated control group (Figure 1A, 1B). Moreover, individual treatment with either PRI-724 or PD-L1 Ab had no anti-tumor effect as these treatments failed to reduce liver weight or Ki67-positive area. However, in contrast, the combination treatment with both agents significantly reduced liver weight and Ki67-positive area (Figure 1A, 1B). These results suggested that the co-administration of PRI-724 and PD-L1 Ab was able to exert an anti-tumor effect on SL4 cell metastasis to the liver. Consistent with these data, the combination therapy also improved the survival rate after the inoculation of colon cancer cells (Figure 1C). Importantly, the combination therapy did not increase serum alanine aminotransferase (ALT) levels (Figure 1D), indicating that there was less adverse effect on hepatocytes.

### PRI-724 treatment reduced mRNA expression of β-catenin target genes in SL4-inoculated livers

**Figure 2:**
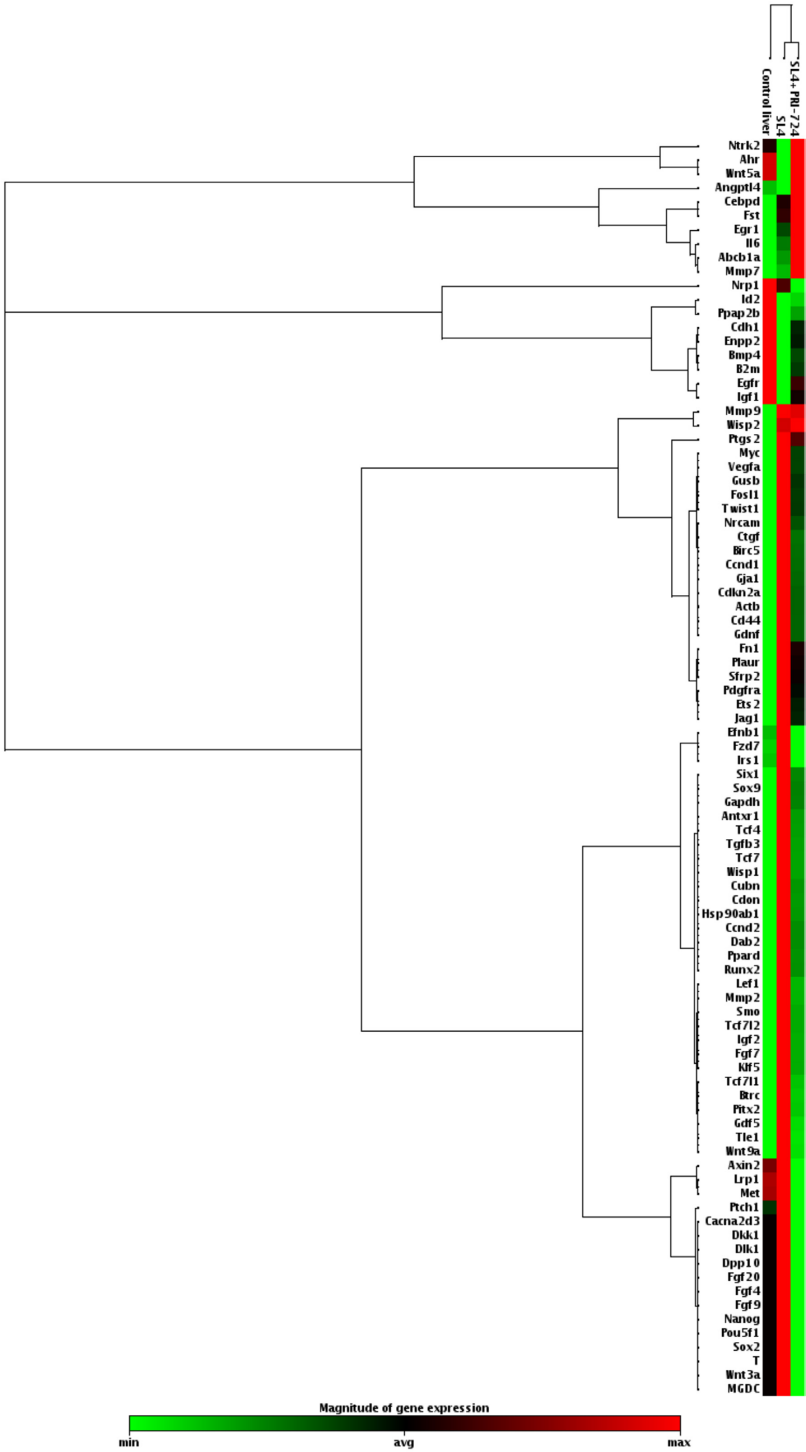
PRI-724 treatment decreased the mRNA expression of β-catenin target genes in the livers of mice inoculated with SL4 cells. After male C57BL/6J mice were intrasplenically injected with SL4 cells (5 × 10^5^ cells) and treated with PRI-724 (0.4 mg/mouse) or PBS three times per week, the animals were humanely sacrificed 14 days after inoculation. Expression of the indicated mRNA expression levels of β-catenin target genes in the liver was determined using an RT^2^ Profiler^™^ PCR Array.

To examine whether Wnt/β-catenin signaling was activated in livers of mice inoculated with SL4 cells, we analyzed the expression levels of Wnt/β-catenin target genes (Figure 2). Inoculation of SL4 cells resulted in increased expression of Wnt/β-catenin target-genes in the livers of mice, which was decreased following PRI-724 treatment, indicating that PRI-724 could inhibit β-catenin signaling in the metastatic liver. These results suggest that effective tumor regression after anti-PD-L1 Ab administration required CBP/β-catenin inhibition in the metastatic liver tumors of colon cancer.

### PRI-724 increased T-lymphocyte infiltration into metastatic liver tumors

**Figure 3:**
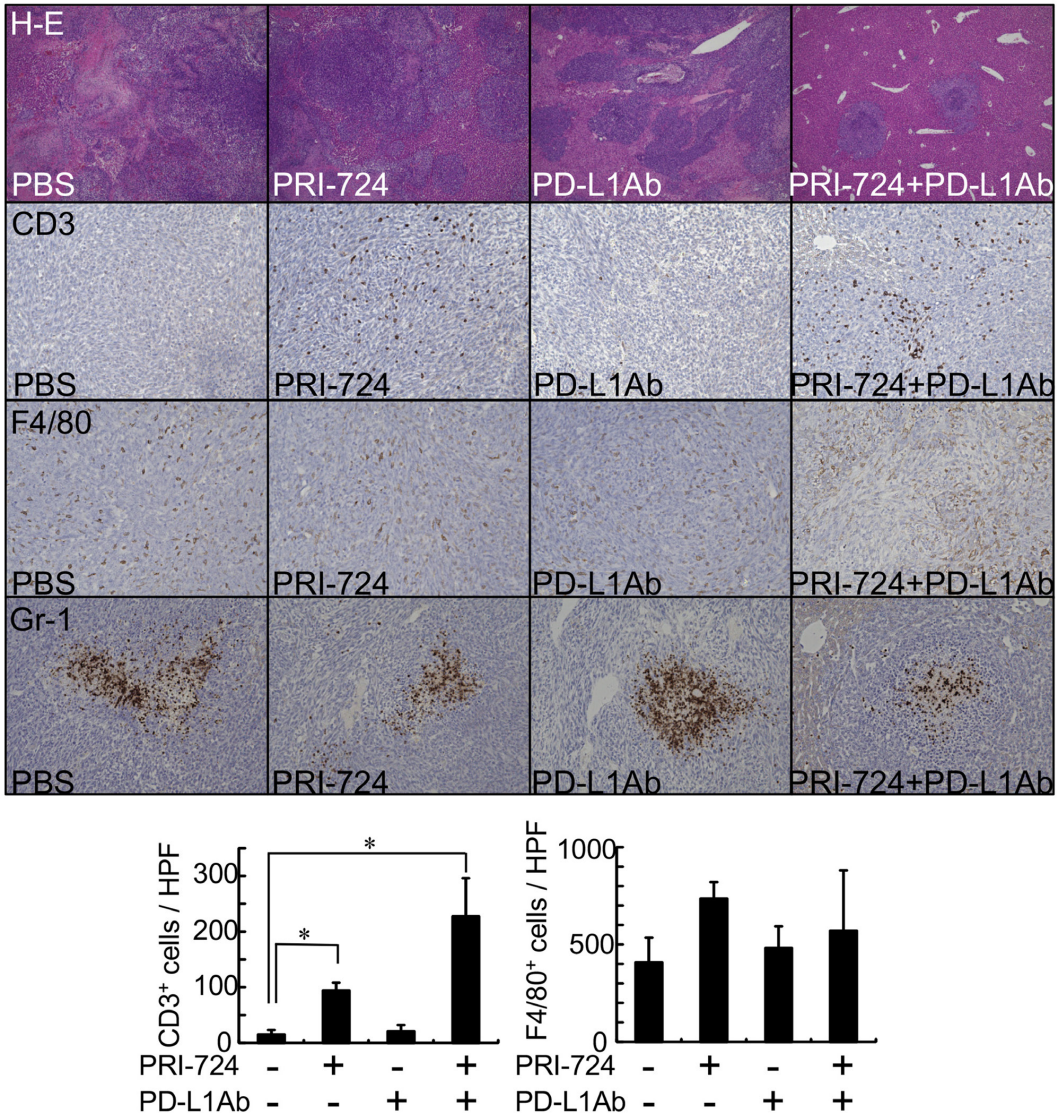
CBP/β-catenin inhibition increased lymphocyte infiltration to metastatic tumors in the liver. Male C57BL/6J mice were intrasplenically injected with SL4 cells (5 × 10^5^ cells). The animals were treated with or without anti-PD-L1 antibody (Ab) and/or PRI-724 and were humanely sacrificed 14 days after inoculation. Liver sections were stained with H&E (original magnification: 40×). Expression of CD3, F4/80, and Gr-1 in the metastatic tumor was examined by immunohistochemistry with an anti-CD3, anti-F4/80, and anti-Gr-1 Abs (graph on lower panel) to assess the number of lymphocytes, macrophages, and neutrophils, respectively (original magnification: 200×). Results are provided as means ± SD of data collected from at least four independent experiments. ^*^P < 0.05 based on one-way ANOVA test.

In melanoma, tumor regression after PD-1 blockade requires pre-existing CD8^+^ T-cells in the tumor [5]. Immunohistochemical analysis was performed using SL4-inoculated liver tissue 14 days after PRI-724 administration to determine whether CD3^+^ T-cells would invade the tumor site. The mean number of CD3^+^ cells per high-power microscopic field in metastatic liver tumors was comparable to that observed in the normal control livers (12 ± 3.5 vs. 15 ± 7.9 cells/high power field, respectively). However, the number of CD3^+^ cells in the tumors increased following PRI-724 treatment (Figure 3). Flow cytometric analysis of isolated intrahepatic leukocytes (IHLs) at 14 days after injection revealed that the relative percentages of CD4^+^, CD8^+^, and NK1.1^+^ cells were not altered by PRI-724 treatment with or without anti-PD-L1 Ab administration (Table 1, Supplementary Figure 2), suggesting that the subsets of T lymphocytes were not altered. The increased number of T lymphocytes observed in the PRI-724-treated group consisted of CD8^+^ T-cells and CD8^+^ T-cells, as well as other subsets of T lymphocytes. Since PRI-724 monotherapy failed to suppress tumor growth, the increase in CD8^+^ T-cell infiltration alone was not sufficient to induce tumor regression. PD-1 is expressed at low levels on naïve T-cells and is upregulated during their activation [13]. In addition, activation of Wnt/β-catenin signaling maintains T cells in an undifferentiated state [14]. The proportion of CD44^−^CD62L^+^ naïve T-cells in among IHLs was not increased by PRI-724 treatment (Table 1), suggesting that the tumor lymphocyte population, which was found to increase, contained PD-1-expressing cells. Furthermore, the anti-PD-L1 Ab increased the percentage of CD69^+^ lymphocytes, indicating the activation of these cells (Table 1). Based on the previous reports and our current findings, the increase in CD8^+^ T-cell infiltration mediated by PRI-724 and their activation induced by anti-PD-L1 Ab are both required for the regression of liver tumors caused by metastatic colon cancer.

**Table 1:** Changes in lymphocyte surface marker profiles in SL4 cell-inoculated mice

Markers	SL4			
	PBS	PRI-724	PD-L1Ab	PRI-724 + PD-L1Ab
CD3^+^ CD4^+^	4.33 ± 1.35	5.57 ± 2.51	5.27 ± 1.54	4.98 ± 1.13
CD3^+^ CD8^+^	7.18 ± 1.64	10.30 ± 3.64	8.30 ± 1.32	8.15 ± 1.66
CD3^+^ NK1.1^+^	2.49 ± 0.62	1.82 ± 0.63	1.85 ± 0.18	2.47 ± 0.64
CD4^+^ CD44^-^ CD62L^+^	46.73 ± 5.80	51.13 ± 6.67	38.8 ± 5.11	37.3 ± 7.88^*^
CD8^+^ CD44^-^ CD62L^+^	37.22 ± 1.85	40.58 ± 9.14	28.15 ± 3.30^*^	30.11 ± 7.76
CD4^+^ CD44^-^ CD62L^-^	10.22 ± 1.82	11.60 ± 2.15	10.80 ± 1.29	10.89 ± 3.29
CD8^+^ CD44^-^ CD62L^-^	9.25 ± 4.58	11.64 ± 2.09	10.55 ± 1.43	8.51 ± 2.71
CD4^+^ CD69^+^	1.06 ± 0.40	1.20 ± 0.26	1.67 ± 0.71	1.89 ± 0.57^*^
CD8^+^ CD69^+^	1.25 ± 0.26	1.69 ± 0.26	2.36 ± 0.21^*^	1.80 ± 0.30^*^
CD4^+^ Foxp3^+^	0.05 ± 0.05	0.11 ± 0.05	0.24 ± 0.14^*^	0.46 ± 0.18^*^

After C57BL/6J male mice were intrasplenically injected with SL4 cells (5 × 10^5^ cells) and treated with either PRI-724 (0.4 mg/mouse, three times per week) or an anti-PD-L1 Ab (200 μg/mouse, three times per week), intrahepatic leukocytes **(**IHLs) were isolated from the median lobe of the livers. The expression of the indicated cell-surface markers was determined by FACS analysis (%). ^*^P < 0.05 versus SL4 cell-inoculated, PBS-treated mice based on a one-way ANOVA or a Kruskal-Wallis rank sum test.

### Role of T cells in the anti-tumor effect in liver

**Figure 4:**
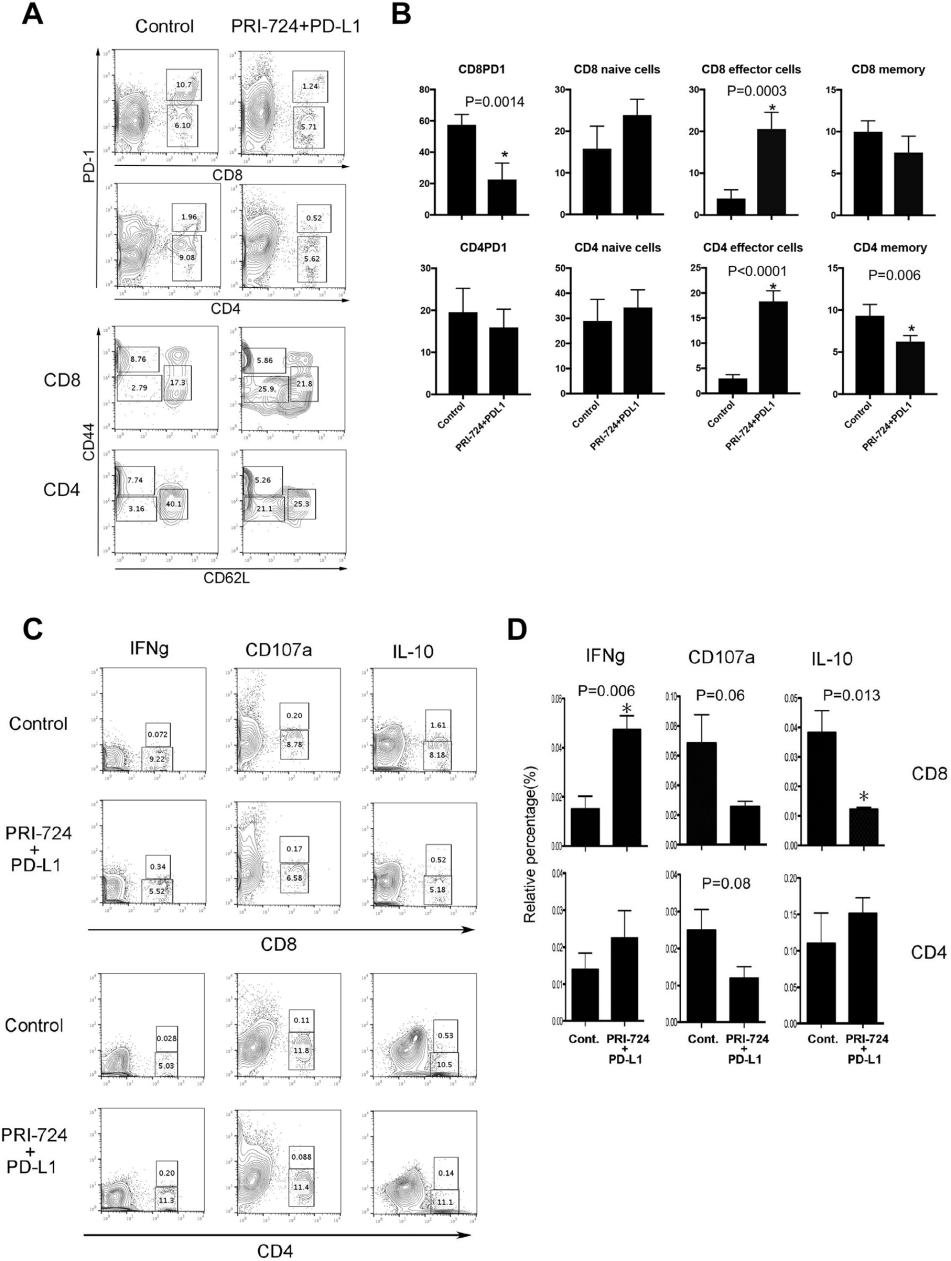
Role of T cells in the anti-tumor effect of anti-PD-L1 antibody (Ab) and/or PRI-724. Male C57BL/6J mice were intrasplenically injected with SL4 cells (5 × 10^5^ cells). The animals were treated with or without anti-PD-L1 Ab and PRI-724 and were humanely sacrificed 5 days after inoculation. (**A**) Intrahepatic leukocytes **(**IHLs) isolated from mice inoculated with SL4 cells were stained for PD-1, CD44, and CD62L in CD8 and CD4 T-cells. The numbers represent the percentage of PD-1-, CD44-, and CD62L-positive and negative cells among the CD8 and CD4 T-cells. (**B**) Results are representative of three independent experiments. Significant relationships are indicated by *P*-values based on a two-tailed Student’s *t*-test. ^*^P < 0.05. (**C**) The numbers represent the percentage of IFN-γ-, CD107a-, and IL-10-positive and negative cells among CD8 and CD4 T-cells. (**D**) Results are representative of three independent experiments. Significant relationships are indicated by *P*-values based on a two-tailed Student’s *t*-test. ^*^P < 0.05.

Because we found that PRI-724 and PD-L1 Ab could induce the recruitment of CD3^+^ T-cells to the liver at 14 days post treatment, we next assessed which T-cell population was essential for the anti-tumor effects. Although results for IHLs were observed at 14 days after PRI-724 administration, it was possible that the immune response to the tumor had already peaked by this time point. Therefore, IHLs were collected and analyzed 5 days after co-administration of PRI-724 and PD-L1 Ab, which is the time at which tumor is found to exist in the liver. IHLs isolated from SL4 cell-inoculated mice were stained with Abs against PD-1, CD44, and CD62L and the effect of the combined treatment was evaluated. The expression of PD-1 on CD8^+^ T-cells was significantly decreased in the combined-treatment group compared to that in the control group. In addition, the proportion of effector CD44^low^CD62L^low^ cells was significantly increased among the CD4^+^ and CD8^+^ T-cells in the liver (Figure 4A, 4B).

Next, to determine the functional activity of CD4^+^ and CD8^+^ T-cells, we performed a CD107a mobilization assay and intracellular interferon (IFN)-γ staining. IHLs isolated from mice inoculated with SL4 cells were co-cultured with SL4 cells and recombinant IL-2 to analyze the cytokine/chemokine production. Cytolytic cell activation was then measured based on the degranulation marker CD107a. As shown in Figure 4C and 4D, the ratio of CD4^+^ and CD8^+^ CD107a^+^-cells relative to all T-cells decreased in IHLs isolated from SL4-inoculated mice after PRI-724 and PD-L1 Ab treatment. This indicated that cytolytic activity, such as perforin and granzyme B release, was not involved in the anti-tumor effects. In contrast, the ratio of CD8^+^ cells, but not CD4^+^IFN-γ^+^ cells, relative to all T cells following PRI-724 and PD-L1 Ab treatment was significantly increased compared to that in control treated mice, suggesting that CD8^+^ cells were activated in the livers of mice treated with the combination therapy. Interestingly, the proportion of CD8^+^ interleukin (IL)-10^+^ cells was also significantly reduced compared to that in the control group after co-injection. Together, these findings indicated that PRI-724 and PD-L1 Ab treatment resulted in the induction of an effective CD8^+^ T-cell immune response against SL4 cells in the liver.

### CD8^+^ T-cells were required for the anti-tumor effect of combined anti-PD-L1 Ab and PRI-724 treatment

**Figure 5:**
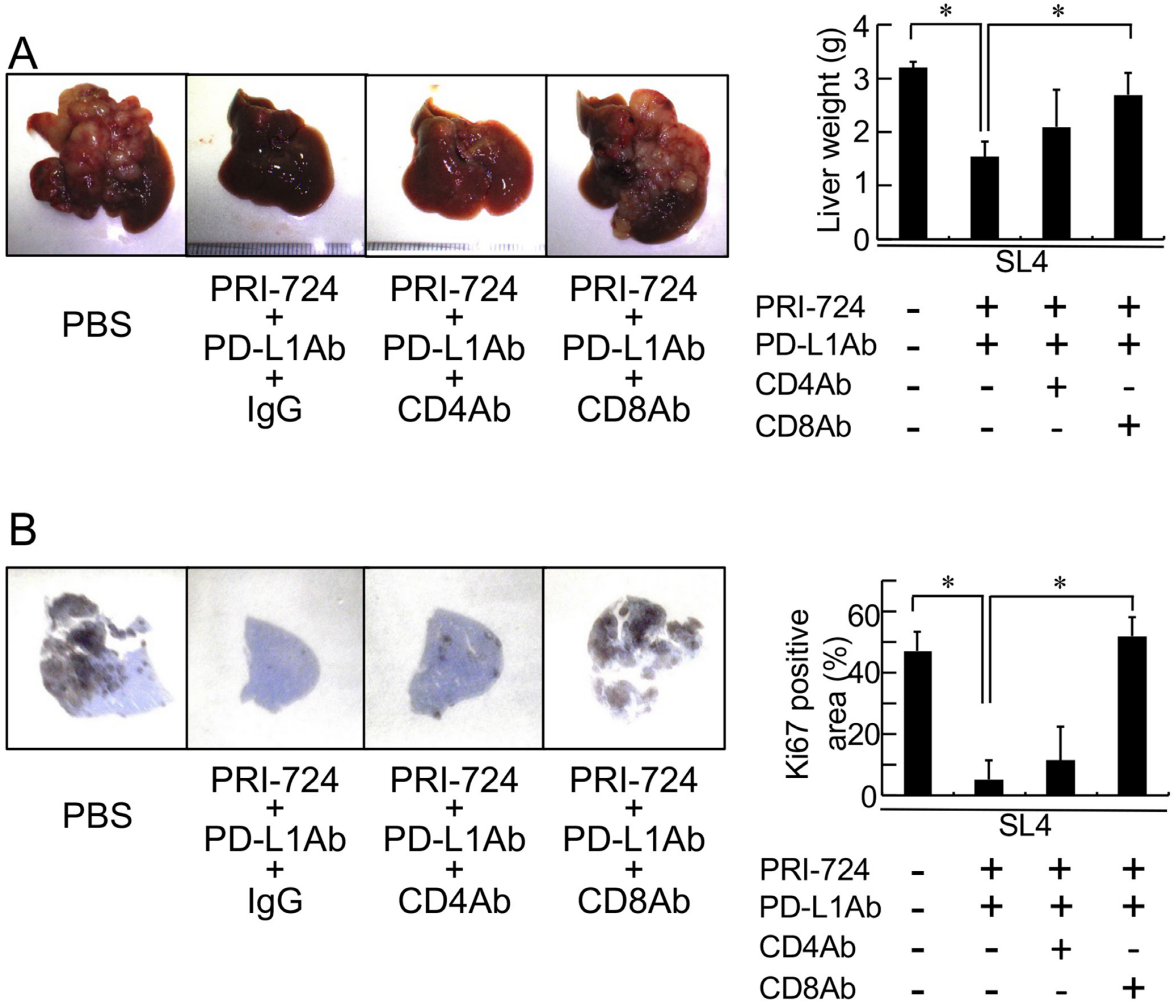
Anti-CD8 antibody (Ab) mitigated the inhibitory effects of an anti-PD-L1 antibody combined with a CBP/β-catenin inhibitor on tumor growth in the liver. Male C57BL/6J mice were intrasplenically injected with SL4 cells (5 × 10^5^ cells). The animals were treated an anti-PD-L1 Ab and PRI-724 with or without an anti-CD4 or anti-CD8 Ab and were humanely sacrificed 14 days after inoculation. (**A**) The livers were excised and photographed (left panels). Liver weights were measured (right panel). (**B**) Expression of Ki67 in the metastatic liver tumor (loupe magnification) was examined by immunohistochemistry using an anti-Ki67 Ab. Intrahepatic tumor load is presented as Ki67-positive area for each animal (graph on right panel). The pictures shown are representative of at least four independent experiments. Results are given as means ± SD of data collected from at least four independent experiments. ^*^P < 0.05 based on a one-way ANOVA test.

To evaluate the role of CD8^+^ T-cells in the anti-tumor effect of the combination treatment, a neutralizing Ab against CD8^+^ T-cells was injected into SL4-inoculated mice prior to administering the PRI-724 and PD-L1 Ab combination treatment. As shown in Figure 5, the anti-CD8 Ab, which eliminated CD8^+^ cells in the tumor (Supplementary Figure 3), inhibited the reductions in liver weight and Ki67-positive area that was mediated by the combined treatment. In contrast, based on liver weight and Ki67-positive area, an anti-CD4 Ab did not alter the anti-tumor effect. These results suggested that the anti-tumor activity of combined PRI-724 and anti-PD-L1 Ab treatment was dependent on CD8^+^ T-cells.

### Inflammatory cytokine and chemokine expression in the livers of SL4-inoculated mice

Activation of β-catenin induces the expression of the transcriptional repressor ATF3, which suppresses CCL4 production leading to T-cell exclusion [6]. Indeed, mRNA expression levels of various chemokines in the livers of SL4-inoculated mice were increased following PRI-724 treatment (Table 2). In addition, serum levels of several chemokines in SL4-inoculated mice were also increased by PRI-724 administration (Supplementary Table 1). *ATF3* mRNA expression was increased *in vitro* in SL4 cells cultured with C-82, an active form of PRI-724 (Supplementary Table 2). However, the expression levels of these chemokines in SL4 cells were low (Supplementary Table 1). Especially for levels of serum CCL4, which were not increased by SL4 cell inoculation or PRI-724 treatment (Supplementary Table 1). In contrast, mRNA expression levels of several chemokines were increased in IHLs isolated from the PRI-724-treated mice (Supplementary Table 3), suggesting that the source of the chemokines was not likely to be SL4 cells, but rather inflammatory cells. These increases in chemokines may have induced the CD8^+^ cells in the tumors.

**Table 2:** Changes in the mRNA profiles of chemokines, cytokines, and biomarkers of classically activated and regulatory macrophages in SL4 cell-inoculated mice

	PBS	PRI-724	SL4	
			PBS	PRI-724
CCL2	1.00 ± 0.59	5.21 ± 1.74	14.15 ± 6.73	28.41 ± 11.47^*^
CCL3	1.00 ± 0.49	4.10 ± 2.23	18.12 ± 8.30	35.98 ± 5.81^*^
CCL4	1.00 ± 0.51	2.48 ± 0.69	11.05 ± 5.50	27.96 ± 10.63^*^
CCL5	1.00 ± 0.57	0.82 ± 0.13	2.40 ± 1.59	3.41 ± 1.06
CCL7	1.00 ± 0.36	8.03 ± 1.80	28.44 ± 14.36	49.65 ± 21.70
CCL8	1.00 ± 1.11	0.41 ± 0.21	10.59 ± 6.66	16.66 ± 6.19
CXCL1	1.00 ± 0.47	18.84 ± 6.51	1.28 ± 1.03	2.21 ± 1.63
CXCL2	1.00 ± 0.24	5.05 ± 2.63	30.99 ± 22.82	39.03 ± 17.59
CXCL3	1.00 ± 0.41	5.08 ± 2.33	58.41 ± 49.23	76.54 ± 48.01
CXCL9	1.00 ± 0.41	1.21 ± 0.09	1.30 ± 0.65	7.57 ± 1.32^*^
CXCL10	1.00 ± 0.37	2.04 ± 0.24	0.66 ± 0.19	3.44 ± 1.02^*^
CXCL12	1.00 ± 0.18	0.81 ± 0.13	0.32 ± 0.09	0.32 ± 0.13
CXCL13	1.00 ± 0.16	1.23 ± 0.14	1.15 ± 0.71	0.25 ± 0.67
TNF-α	1.00 ± 0.47	2.38 ± 0.71	3.11 ± 1.57	8.09 ± 1.62^*^
IL-1β	1.00 ± 0.26	2.65 ± 1.05	3.19 ± 1.84	8.87 ± 2.20^*^
IL-6	1.00 ± 0.64	3.66 ± 1.63	10.59 ± 4.41	21.79 ± 15.09
CD11c	1.00 ± 0.68	0.53 ± 0.16	31.64 ± 12.49	85.24 ± 30.64^*^
IFN-γ	1.00 ± 1.31	0.47 ± 0.04	3.12 ± 4.20	31.52 ± 17.98^*^
CD163	1.00 ± 0.24	1.07 ± 0.10	1.13 ± 0.59	0.77 ± 0.35
Mannose receptor	1.00 ± 0.48	0.80 ± 0.34	1.00 ± 0.36	1.26 ± 0.64
IL-10	1.00 ± 0.70	1.96 ± 1.01	26.57 ± 12.50	31.93 ± 7.84

After C57BL/6J male mice were intrasplenically injected with SL4 cells (5 × 10^5^ cells) and treated with either PRI-724 (0.4 mg/mouse, three times per week) or PBS, the animals were humanely sacrifice 14 days after inoculation. Expression of the indicated mRNA in the liver was determined by quantitative real-time RT-PCR. Results are presented as means ± SD of data collected from at least four independent experiments. ^*^P < 0.05 versus SL4 cell-inoculated, PBS-treated mice based on a two-tailed Student’s *t*-test.

## DISCUSSION

In the current study, we investigated the effect of a CBP/β-catenin inhibitor combined with PD-L1/PD-1 blockade on the suppression of tumor growth in a mouse model of colon cancer liver metastasis. Our results indicated that inhibition of CBP/β-catenin increased the therapeutic effect of PD-L1/PD-1 blockade through the accumulation of CD8^+^ T-cells in the tumors. These results suggest therapeutic potential for treating metastatic liver tumors derived from colon cancer.

Previously, poor anti-tumor efficacy was reported for anti-PD-1/PD-L1 monotherapies with colorectal cancer [2, 4]. Similarly, anti-PD-L1 Ab monotherapy demonstrated no anti-tumor activity in our mouse model. However, an inverse correlation between PD-L1 expression and disease-free survival in stage III colorectal cancer has been reported [15]. Therefore, the immune checkpoint appears to contribute to colon cancer progression. Importantly, *in vitro* evaluation of SL4 cells treated with C-82 and an anti-PD-L1 Ab revealed no cytotoxic effects (data not shown). In melanoma, tumor regression mediated by the anti-tumor effects of an anti-PD-1 Ab requires the presence of CD8^+^ T-cells in the tumor [5]. In colon cancer, tumor T-lymphocytes correlate with patient survival [16–18] and enhanced CD8^+^ T-cell infiltration to the invasive margin of liver metastasis is predictive of a better response to chemotherapy [19]. In our model, the number of CD3^+^ cells in metastatic tumors was comparable to that in normal liver tissues. Moreover, anti-PD-L1 Ab monotherapy did not increase the number of CD3^+^ cells. In such states of diminished T-lymphocytes in tumors, the anti-tumor effects of the anti-PD-L1 Ab might not be apparent. In contrast, the number of CD3^+^ cells did increase in metastatic tumors following PRI-724 treatment and anti-tumor activity by anti-PD-L1 Ab was demonstrated in these mice. These anti-tumor effects were mitigated by an anti-CD8^+^ Ab, indicating that an increase in CD8^+^ cells was essential for the anti-tumor activity of the anti-PD-L1 Ab in this model. Because PRI-724 monotherapy failed to show anti-tumor effects, despite the increase in CD3^+^ cells, T-cell accumulation was essential but not sufficient for the inhibition of metastatic tumor growth. Following T-cell activation, PD-1 is expressed and receptor signals limit the expansion and activation of T-cell receptor-triggered T cells [20]; indeed, anti-PD-L1 therapy increased lymphocyte activation in our model. These results suggest that, in addition to the increase in T-cell infiltration mediated by PRI-724, T-cell activation via anti-PD-L1 agents was required for anti-tumor activity. Moreover, the accumulation of T cells in the tumors was more evident in animals treated with the combination of these drugs (Figure 3). Furthermore, the combined treatment lowered expression of PD-1 on CD8^+^ T-cells in the liver, which resulted in infiltration of effector CD44^low^CD62L^low^ cells among the CD4^+^ and CD8^+^ T-cells. Consistent with this result, the CD8^+^ T-cells in the liver exhibited enhanced IFN-γ production, and conversely, IL-10 production was suppressed when CD8^+^ T-cells were co-culture with SL4 cells. These findings revealed that the combined treatment induced a robust CD8^+^ T-cell immune response to the tumor. The anti-tumor activity exerted by CD8^+^ T-cells was not a result of the degranulation of CD8^+^ cells, and thus, apoptosis pathways via TNF-α or Fas may have been involved in the anti-tumor effects. Unlike that observed in melanoma, the accumulation of T lymphocytes was not due to the production of CCL4 by the tumors. Instead, the levels of several chemokines were increased in IHLs isolated from PRI-724-treated mice. We previously reported that the depletion of liver macrophages via alendronate liposome treatment increases SL4 cell tumor growth in the liver, which is accompanied by a decrease in CD3^+^ cells in metastatic liver tumors [21], suggesting that liver macrophages promote T-cell accumulation.

It has been demonstrated that liver macrophages modulate the host immune response to cancer cells by releasing cytotoxic products and immune-stimulating factors [22]. Macrophage depletion increases the severity of tumor growth in the livers of SL4-inoculated mice [21], which is accompanied by a decrease in CD3^+^ T-cell infiltration. Although the number of F4/80^+^ cells per high-power microscopic field of metastatic liver tumors was not altered (Figure 3), the expression of inflammatory cytokines TNF-α and IL-1β, which are mainly produced by macrophages in the liver, was increased in the livers of SL4-inoculated mice following PRI-724 treatment (Table 2). In addition, TNF-α serum levels were increased by PRI-724 treatment in these animals (Supplementary Table 2). Moreover, mRNA expression levels of the classically activated M1 macrophage markers *CD11c* and *IFN-γ* were increased by PRI-724 treatment, whereas expression levels of the genes encoding markers of regulatory M2 macrophages, such as CD163, mannose receptor, and IL-10, were not affected (Table 2). M1 macrophages are considered to be cytotoxic macrophages that inhibit cancer growth while repair-type M2 macrophages stimulate tumor growth [23]. Thus, PRI-724 also appears to contribute to tumor regression by altering macrophage properties and promoting a cytotoxic phenotype, which might induce T-cell infiltration to the tumor. In an autoimmune hepatitis mouse model induced by neonatal thymectomy and PD-1 knockout, the migration of T cells was found to be triggered by hepatic macrophages that produce c-x-c motif ligand (CXCL) 9 [24]. M1 and M2 macrophages have different chemokine-production profiles with M1 macrophages thought to produce T-cell-attracting chemokines such as CXCL9 and CXCL10 [25]. In our model, it appeared that PRI-724 altered macrophage properties toward a cytotoxic M1 phenotype since *CXCL9* and *CXCL10* mRNA expression were increased by PRI-724 in the livers of mice inoculated with SL4 cells. In addition to macrophages, dendritic cells also express CD11c and may also contribute to the activity. Thus, we believe that the accumulation of T lymphocytes caused by PRI-724 might occur through M1 macrophages and dendritic cells. This is supported by the fact that β-catenin is well known to contribute to macrophage motility and adhesion [26]; however, its roles in chemokine production in this model remains unclear.

In contrast, Gr-1-positive cells accumulated locally in the central part of the tumor (Figure 3), suggesting that neutrophils did not contribute to the observed tumor regression. Regulatory T (Treg) lymphocytes are known to inhibit anti-tumor immunity by inhibiting the effector T-cell response. Overexpression of stabilized β-catenin in Treg lymphocytes was previously found to increase the survival of these cells [27]. However, Treg lymphocytes in our study were not decreased by treatment with PRI-724 and anti-PD-L1 Ab (Table 1), suggesting that these cells were not involved in the observed anti-tumor effects.

Unfortunately, our data failed to identify the source of chemokines found to be induced upon PRI-724 treatment; however, the effect of the anti-PD-L1 Ab on macrophages has not yet been investigated. It is not clear which chemokines were involved in the PRI-724-induced CD8^+^ cell infiltration into the tumors. Moreover, the potential roles of other liver cells currently remains unclear as does the mechanism through which CBP/β-catenin stimulates the activation of macrophages. The effects of combination therapy were not sufficient to induce the complete elimination of the tumors. It is clear that further studies are needed to resolve these uncertainties.

In conclusion, inhibition of CBP/β-catenin increased the anti-tumor effects of anti-PD-L1 Ab therapy against metastatic liver tumors derived from colon cancer by promoting the accumulation of CD8^+^ T-cells and inducing a shift in the properties of macrophages toward a cytotoxic phenotype. Thus, approaches targeting CBP/β-catenin combined with PD-1/PD-L1 immune checkpoint blockade represents a novel therapeutic strategy for treating liver metastasis during colon cancer.

## MATERIALS AND METHODS

### Study approval

The experiments were conducted in accordance with institutional guidelines (Guide for the Care and Use of Laboratory Animals prepared by the National Academy of Sciences) and the protocol was approved by the Research Committee of Komagome Hospital. All surgery was performed under anesthesia and all efforts were made to minimize suffering.

### Information regarding PRI-724 and C82

C-82 is a second-generation specific CBP/β-catenin antagonist developed by Prism Pharma, which inhibits the binding between β-catenin and CBP and increases the binding between β-catenin and p300. PRI-724 is the phosphorylated form of C-82 and is rapidly hydrolyzed *in vivo* to its active form C-82. The chemical name of PRI-724 is 4-(((6S,9S,9aS)-1-(benzylcarbamoyl)-2,9-dimethyl-4,7-dioxo-8-(quinolin-8-ylmethyl)octahydro-1H-pyrazino[2,1-c][1,2,4]triazin-6-yl)methyl)phenyldihydrogen phosphate. The chemical name of C-82 is 4-(((6S,9S,9aS)-1-(benzylcarbamoyl)-2,9-dimethyl-4,7-dioxo-8-(quinolin-8-ylmethyl)octahydro-1H-pyrazino[2,1- c][1,2,4]triazin-6-yl)methyl)phenyl.

### Cell culture

A green fluorescent protein (GFP)-expressing SL4 mouse colon adenocarcinoma cell line (Anti-Cancer Japan, Osaka, Japan) was maintained as a monolayer culture in RPMI-1640 medium (Invitrogen, Carlsbad, CA, USA) containing 10% fetal bovine serum (FBS) supplemented with penicillin and streptomycin (Invitrogen). For *in vitro* experiments, the cells were treated with or without C-82 (1 μM) for 24 h. For cell inoculation into mice, SL4 cells were harvested using trypsin and EDTA, washed with PBS, and then resuspended in PBS at a concentration of 5 × 10^6^ cells/mL.

### Liver metastasis model

Male wild-type C57BL/6J mice 8-weeks of age were obtained from Japan SLC (Shizuoka, Japan). After making a small incision under anesthesia to expose the spleen, 0.1 mL of a viable cell suspension containing 5 × 10^6^ cells/mL was injected into the spleen. We chose SL4 cells because the cells grow rapidly, even in the liver of wild-type mice [21]. The animals were then each intraperitoneally injected with or without 0.4 mg PRI-724 (Prism Pharma, Yokohama, Japan) and/or 200 μg of an anti-PD-L1 Ab (10F.9G2; Bio X Cell, NH, USA) three times per week. In addition, some mice treated with PRI-724 and the anti-PD-L1 Ab were administrated anti-mouse CD4 or CD8 Ab (250 μg/mouse; Bio X Cell) three times per week. After the course of treatment, the mice were anesthetized and humanely sacrificed by exsanguination 14 days post cell-inoculation. The livers of the animals were immediately removed, washed in ice-cold PBS, and weighed before a portion of the dissected liver tissue was frozen in liquid nitrogen. Additional animals were maintained and used for survival analysis.

### Histological analysis

The mouse livers were fixed with 10% formalin, sectioned, and stained with hematoxylin and eosin (H-E). Ki67, CD3, F4/80, and Gr-1 immunostaining was performed using anti-Ki67 (SP6, NeoMarkers, Fremont, CA, USA), anti-CD3 (SP7, Abcam, Cambridge UK), anti-F4/80 (Santa Cruz Biotechnology, Santa Cruz, CA, USA), or Gr-1 (eBioscience, San Diego, CA, USA) Abs, respectively, and a VECTASTAIN Elite ABC Kit. Diaminobenzidine tetrahydrochloride (DAB) was used as the peroxidase substrate and sections were counterstained with hematoxylin. The positive immunostained area was analyzed using ImageJ software (National Institutes of Health [NIH], Bethesda, MD, USA; http://rsb.info.nih.gov/ij/) and the percentage of positive-stained tissue relative to the total area of the tissue section was determined.

### Quantitative reverse transcription polymerase chain reaction (RT-qPCR)

RNeasy and DNase Kits from Qiagen (Valencia, CA, USA) were used for RNA extraction from liver tissue and cultured cells, respectively, and a High-Capacity cDNA Reverse Transcription Kit from Applied Biosystems (Foster City, CA, USA) was used for reverse transcription of the RNA into complementary DNA (cDNA). Real time RT-qPCR was performed using the SYBR Premix Ex Taq (Takara, Shiga, Japan) for genes encoding CCL-2, CCL-3, CCL-4, CCL-5, CCL-7, CCL-8, CXCL-1, CXCL-2, CXCL-3, CXCL-9, CXCL-10 CXCL-12, CXCL-13, ATF-3, CD11c, IFN-γ, CD163, mannose receptor, and IL-10 (primer sequences are shown in Supplementary Table 4). Probe and primer sets from Applied Biosystems was used for PCR amplification of 18S rRNA (Hs99999901s1), tumor necrosis factor (TNF)-α (Mm00443258m1), IL-1β (Mm00434228m1), and IL-6 (Mm99999064m1) using Thunderbird Probe qPCR mix (Toyobo, Tokyo, Japan) and a LightCycler 480 (Roche Applied Science, Mannheim, Germany). The results were normalized to 18S rRNA expression values. For mRNA expression analysis of Wnt target genes, an RT^2^ Profiler^™^ PCR Array (Mouse Wnt Signaling Targets, QIAGEN Sciences, MD, USA) was used and real time RT-qPCR was performed using RT² SYBR Green qPCR Mastermix and an RT^2^ First Strand Kit (QIAGEN).

### Isolation of mouse IHLs

Single-cell suspensions were prepared from the liver median lobe by digesting the tissue in RPMI-1640 (FUJIFILM Wako Pure Chemical Corporation, Osaka, Japan) containing 0.02% collagenase IV and 0.002% DNase I (Sigma-Aldrich, St. Louis, MO, USA) for 40 min at 37°C [28]. The cells were overlaid onto Lympholyte-M (Cedarlane, Westbury, NY, USA) in PBS. After density separation, the isolated IHLs were evaluated by FACS analysis.

### FACS analysis

The cells were surface-stained with fluorochrome-conjugated Abs for 20 min on ice using the following Abs: anti-CD3, anti-CD4, anti-CD8, anti-NK1.1, anti-CD69 (eBioscience), and anti-Foxp3 (BioLegend, San Diego, CA, USA). To perform intracellular cytokine staining, IHLs were co-cultured with SL4 cells (1 × 10^5^ cells/well) for 4 h at 37°C in 96-well round-bottom plates containing 200 μL/well RPMI-1640 medium. Mouse recombinant IL-2 (50 units) and 0.2 μL of BD GolgiPlug protein transport inhibitor (BD Biosciences) were added to each well. After incubation, the cells were harvested, washed in PBS containing 1% FBS, and incubated for 10 min on ice with unlabeled anti-mouse CD16/32 Ab (BD Biosciences) to block FcγRII/III binding. The cells were then surface-stained for 20 min on ice with the indicated Abs. Following staining, the cells were washed to remove unbound Ab and fixed using a Cytofix/Cytoperm Kit (BD Biosciences). Cells were then subjected to secondary staining with reagents obtained from BioLegend except as noted, including the following: FITC-conjugated CD107a, PE-conjugated anti-interferon gamma, and anti-IL-10 (BD Biosciences). Data were acquired using a FACSCalibur flow cytometer and analyzed using CellQuest (BD Immunocytometry Systems, San Jose, CA, USA) and FlowJo software (Tree Star, San Carlos, CA, USA).

### Measurement of serum cytokines and chemokines

Serum ALT levels were measured using a Wako Transaminase CII-test Kit (FUJIFILM Wako). Serum cytokines and chemokines were measured using a Luminex MILLIPLEX MAP Mouse Cytokine/Chemokine Magnetic Bead Panel - Immunology Multiplex Assay (MCYTOMAG-70K; Merck Millipore, Darmstadt, Germany). This procedure was performed in accordance with the manufacturer’s instructions.

### Statistical analysis

Results are expressed as the mean ± standard deviation (SD) of the data collected from at least three independent experiments. Data between groups were analyzed by one-way ANOVA, the Kruskal-Wallis test, or a two-tailed Student’s *t*-test. Kaplan–Meier survival analysis was performed using the log-rank test. A *P*-value less than 0.05 was considered statistically significant. The software used for all statistical analyses was Graph Pad Prism version 7 (Graph Pad Software Inc., San Diego, CA, USA).

## SUPPLEMENTARY MATERIALS



## Abbreviations

PD-L1: programmed cell death ligand 1
CREB: cAMP-response element-binding protein
CBP: (CREB)-binding protein
PD-1: programmed cell death 1
IFN: interferon
Ab: antibody
CCL: chemokine ligand
ATF: AMP-dependent transcription factor
IHL: intrahepatic leukocytes
IL: interleukin
CXCL: c-x-c motif ligand
T-reg: regulatory T
TNF: tumor necrosis factor
