# Supplementary material for: Programmed cell death ligand 1 (PD-L1) blockade attenuates metastatic colon cancer growth in cAMP-response element-binding protein (CREB)-binding protein (CBP)/β-catenin inhibitor-treated livers
Source: Oncotarget. 2019 Apr 30;10(32):3013–26. doi: 10.18632/oncotarget.26892 (PMC6508964; doi:10.18632/oncotarget.26892)
Supplement: Supplementary file 1 [file oncotarget-10-3013-s001.pdf]

Programmed cell death ligand 1 (PD-L1) blockade attenuates metastatic colon cancer growth in cAMP-response element-binding protein (CREB)-binding protein (CBP)/β-catenin inhibitor-treated livers

SUPPLEMENTARY MATERIALS

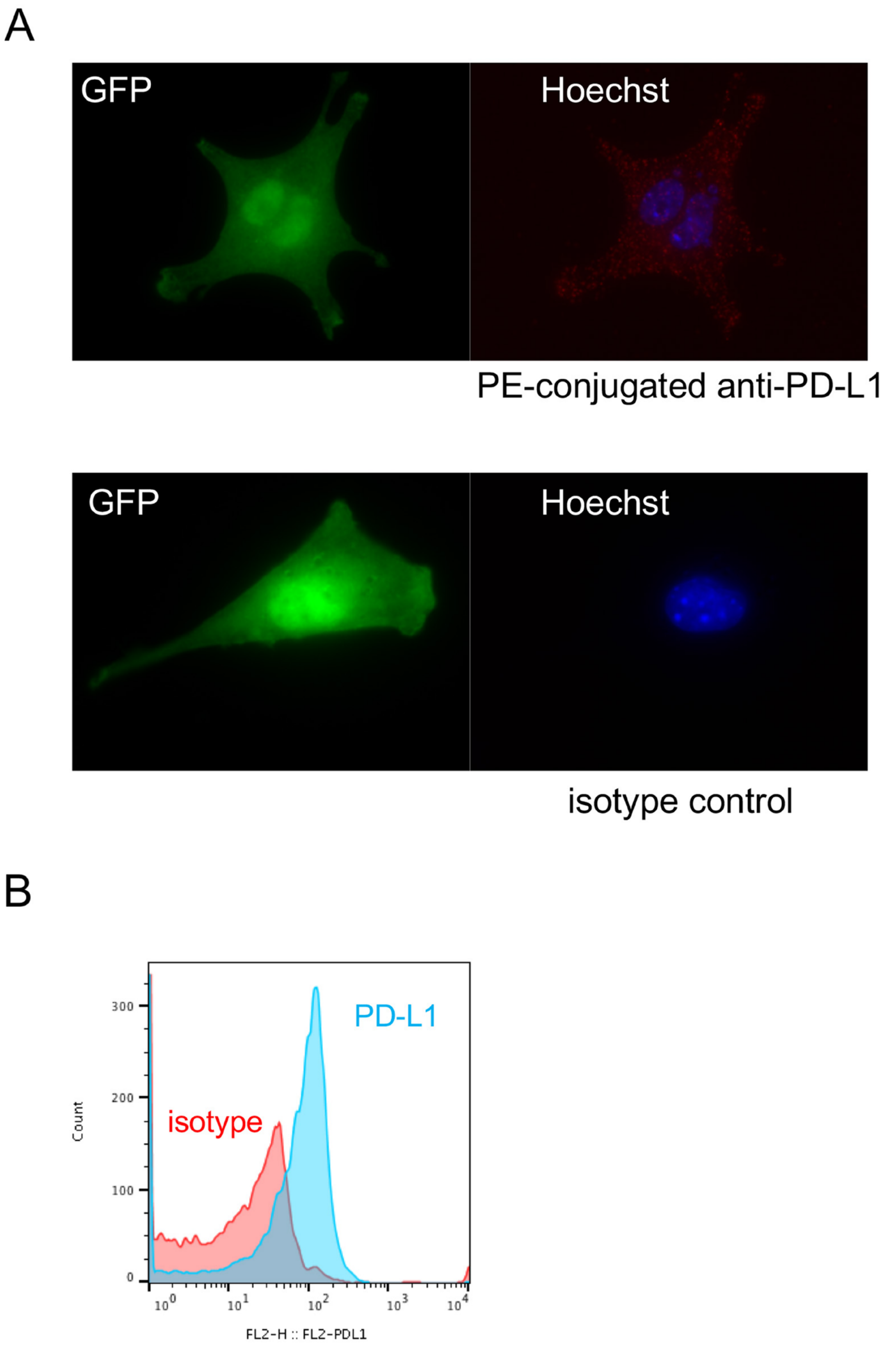

Supplementary Figure 1: SL4 cells express PD-L1. SL4 cells were stained with PE-conjugated anti-PD-L1 antibody (Ab; BioLegend, San Diego, CA, USA, 124308). Merged images with nuclear staining based on Hoechst 33342 (Dojindo, Chiba, Japan, 346-07951) are shown. The pictures shown are representative of at least four independent experiments (**A**). SL4 cells were stained by PE-conjugated anti-PD-L1 Ab (BioLegend) and isotype control. The expression levels of PD-L1 were analyzed by FACS (**B**).

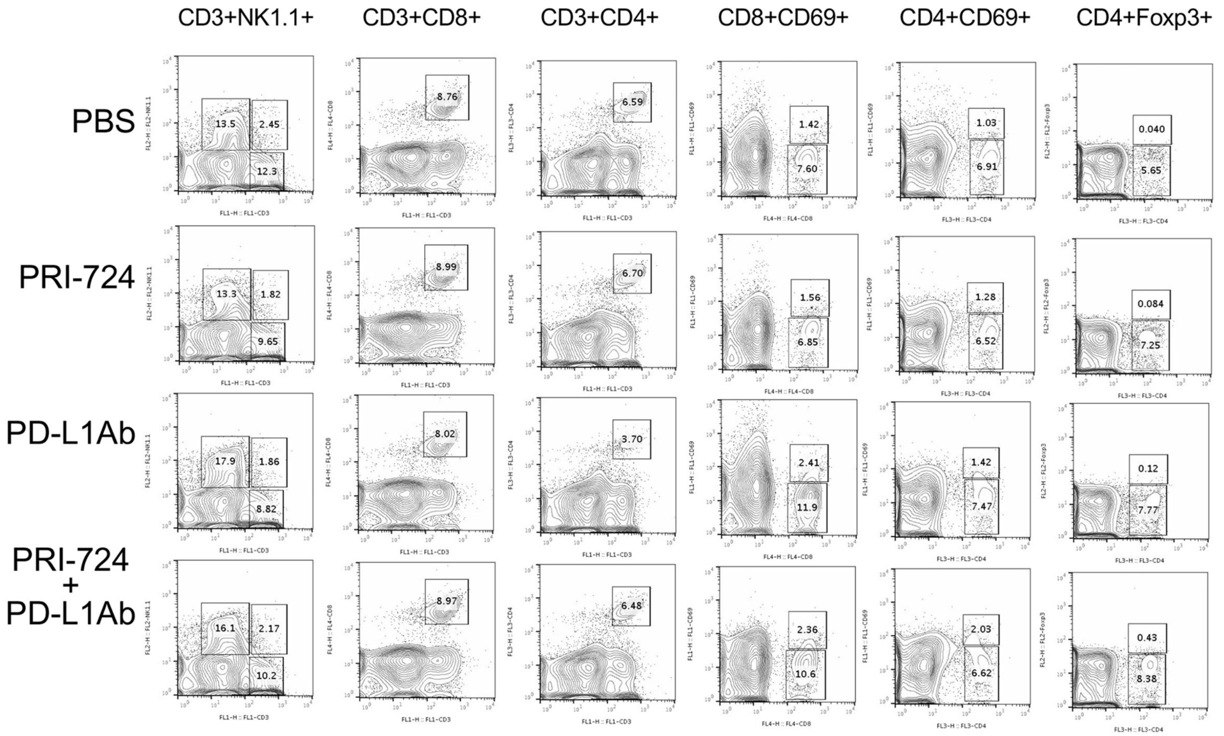

Supplementary Figure 2: Changes in lymphocyte surface marker profiles in mice inoculated with SL4 cells. After male C57BL/6J mice were intrasplenically injected with SL4 cells (5×10^5 cells) and treated with PRI-724 (0.4 mg/mouse) three times per week or an anti-PD-L1 antibody (200 µg/mouse) three times per week, intrahepatic leukocytes (IHLs) were isolated from the liver median lobe. The expression of the indicated cell-surface markers was determined by FACS analysis.

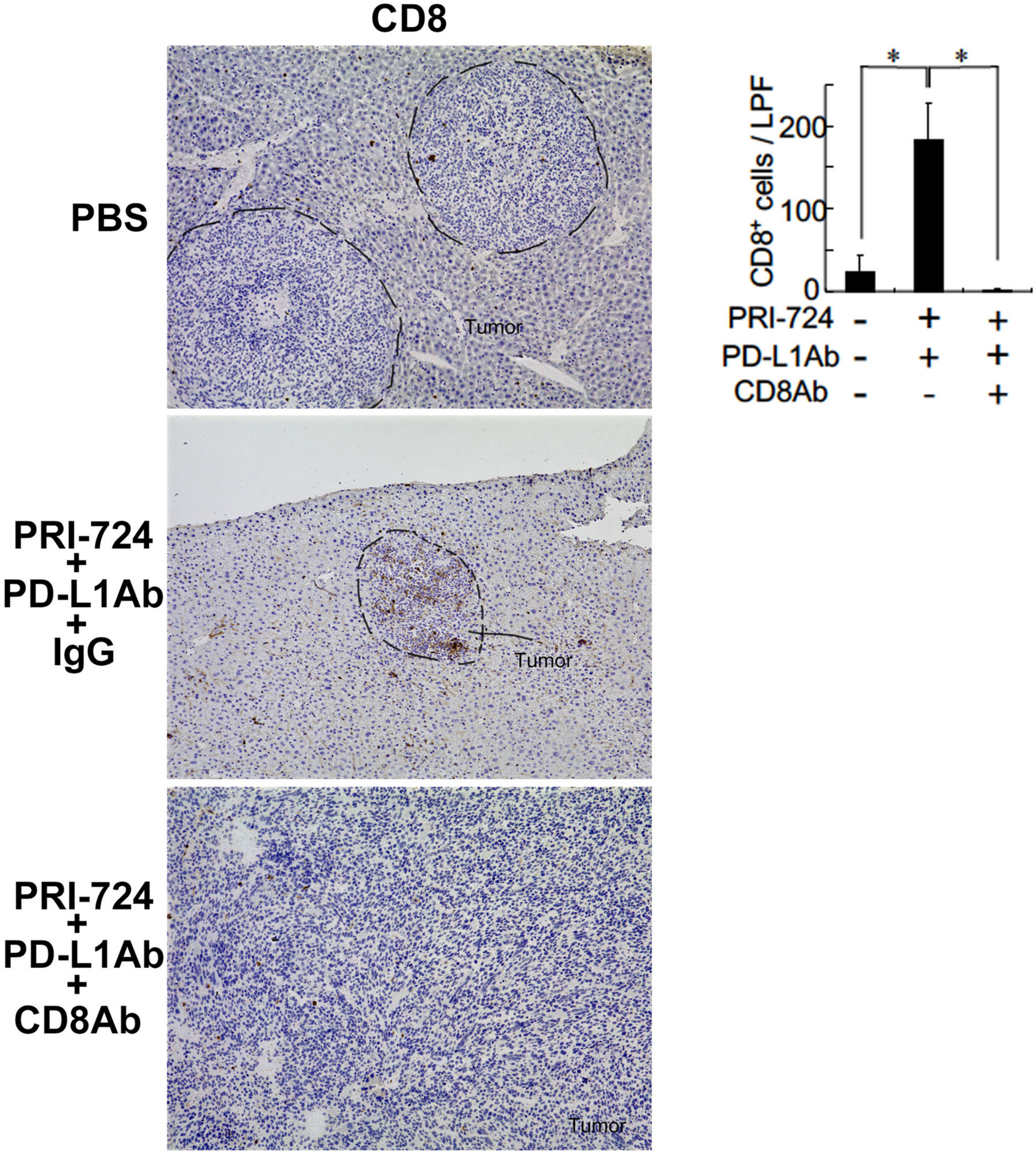

Supplementary Figure 3: Anti-CD8 antibody (Ab) eliminated CD8+ T lymphocytes infiltration to the metastatic tumor in the liver. Male C57BL/6J mice were intrasplenically injected with SL4 cells (5×10^5 cells). The animals were treated with anti-PD-L1 Ab and PRI-724 in conjunction with or without anti-CD8 Ab and were humanely killed 14 d after inoculation. Expression of CD8 in the metastatic tumor was examined by immunohistochemistry with an anti-CD8 Ab (BD Biosciences, 553027) using the frozen sections to assess the number of $CD8^+$ lymphocytes (original magnification: 100×). The number of $CD8^+$ cells in the tumor was counted and adjusted according to the tumor area in the field (100×) (graph on left panel). $^*P < 0.05$ using a one-way ANOVA test.

Supplementary Table 1: Changes in the profiles of serum cytokines and chemokines in SL4-inoculated mice

(pg/mL)	PBS	PRI-724	SL4	
			PBS	PRI-724
G-CSF	697 ± 254	1074 ± 380	1611 ± 1259	2089 ± 1236
CCL11	631 ± 224	1100 ± 178	354 ± 189	422 ± 288
GM-CSF	17.9 ± 4.6	27.6 ± 3.1	12.3 ± 16.9	13.3 ± 5.5
IFN-γ	0.73 ± 0.70	2.17 ± 2.33	1.04 ± 1.59	1.53 ± 1.83
IL-1a	283 ± 117	333 ± 34	134 ± 98	97 ± 59
IL-1b	14.0 ± 6.4	21.5 ± 4.1	16.4 ± 6.3	19.6 ± 5.7
IL-2	0.80 ± 0.63	2.69 ± 0.58	1.60 ± 1.37	1.61 ± 0.88
IL-4	N. D.	N. D.	2.32 ± 1.91	2.39 ± 1.26
IL-3	N. D.	N. D.	N. D.	N. D.
IL-5	5.75 ± 1.93	9.64 ± 1.87	32.7 ± 27.5	70.83 ± 52.5*
IL-6	10.2 ± 3.0	176.6 ± 59.3	67.4 ± 50.4	88.7 ± 46.9
IL-7	1.18 ± 2.04	5.11 ± 6.21	9.21 ± 34.44	0.52 ± 1.48
IL-9	249 ± 41	239 ± 21	290 ± 134	395 ± 456
IL-10	6.2 ± 2.9	13.9 ± 4.0	30.2 ± 23.4	39.8 ± 11.9
IL-12p40	9.7 ± 1.6	12.6 ± 2.9	3.8 ± 2.8	4.6 ± 3.1
IL-12p70	14.8 ± 2.8	13.4 ± 7.2	8.0 ± 7.1	13.5 ± 6.8
LIF	1.86 ± 0.19	4.40 ± 2.03	5.27 ± 2.61	6.86 ± 3.55
IL-13	127 ± 29	153 ± 23	113 ± 30	112 ± 28
CXCL5	9421 ± 1322	10631 ± 1041	4510 ± 2440	3012 ± 2845
IL-15	63.6 ± 38.2	72.1 ± 18.7	14.1 ± 24.8	5.7 ± 10.7
IL-17	6.40 ± 0.47	8.23 ± 2.35	3.12 ± 1.94	2.88 ± 1.98
CXCL10	295 ± 68	287 ± 44	307 ± 65	444 ± 115*
CXCL1	154 ± 57	2390 ± 874	467 ± 411	377 ± 165
CCL2	57.6 ± 13.7	111.9 ± 30.5	67.5 ± 28.6	87.1 ± 18.5
CCL3	78.5 ± 10.3	86.9 ± 5.1	54.2 ± 30.0	68.3 ± 16.3
CCL4	62.0 ± 3.1	99.5 ± 15.3	41.5 ± 25.3	53.0 ± 21.9
M-CSF	16.8 ± 2.0	21.2 ± 4.0	11.6 ± 4.1	15.9 ± 3.4*
CXCL2	171 ± 8	211 ± 28	101 ± 93	93 ± 24
CXCL9	55.5 ± 7.8	55.7 ± 12.1	557.4 ± 217.8	1160.5 ± 672.3*
CCL5	29.8 ± 4.8	35.6 ± 12.7	21.4 ± 8.3	25.5 ± 9.4
VEGF	2.91 ± 0.23	2.90 ± 0.23	2.86 ± 0.66	3.11 ± 1.00
TNF-α	6.83 ± 1.45	11.49 ± 2.62	6.85 ± 2.06	10.06 ± 2.02*

After C57BL/6J male mice had been intrasplenically injected with SL4 (5×10^5 cells) and were treated with PRI-724 (0.4 mg/mouse, three times a week) or PBS, the animals were humanely killed 14 days after inoculation. Serum cytokines and chemokines were determined by MILLIPLEX**.** Results are presented as means ± SD of data collected from at least 4 independent experiments. *$P < 0.05$ versus SL4-inoculated PBS-treated mice using a 2-tailed Student's *t*-test. N.D.: not detected.

Supplementary Table 2: Changes in the mRNA profiles of chemokines and cytokines in SL cells

	DMSO	1 µM C-82
ATF3	1.00 ± 0.09	0.40 ± 0.01*
CCL2	Low expression	
CCL3	Low expression	
CCL4	Low expression	
CCL5	Low expression	
CCL7	Low expression	
CCL8	Low expression	
CXCL1	Low expression	
CXCL2	Low expression	
CXCL3	Low expression	
CXCL9	Low expression	
CXCL10	1.00 ± 0.27	0.99 ± 0.04
CXCL12	1.00 ± 0.16	2.06 ± 0.11*
CXCL13	1.00 ± 0.22	0.51 ± 0.05*

SL4 cells were treated with or without C-82 (1 µM) for 24 hours. Expression of the indicated mRNA variants was determined by quantitative real-time RT-PCR. Results are presented as means ± SD of data collected from at least 4 independent experiments. $^{*}P < 0.05$ versus DMSO-treated cells using a 2-tailed Student's *t*-test. Low expression; the mRNA expression level in SL4 cells was below one fifth of that in SL4 inoculated liver.

Supplementary Table 3: Changes in the mRNA profiles of chemokines and cytokines in PRI-724-treated liver

	PBS	PRI-724
CCL2	1.00 ± 0.69	1.49 ± 0.49
CCL3	1.00 ± 0.46	2.35 ± 0.16*
CCL4	1.00 ± 0.35	1.94 ± 0.26*
CCL5	1.00 ± 055	1.21 ± 0.23
CCL7	1.00 ± 0.72	0.76 ± 0.36
CCL8	1.00 ± 0.55	2.84 ± 2.11
CXCL1	1.00 ± 0.72	0.60 ± 0.11
CXCL2	1.00 ± 0.67	2.40 ± 0.65*
CXCL3	1.00 ± 0.58	2.79 ± 0.85*
CXCL9	1.00 ± 0.65	0.91 ± 0.60
CXCL10	1.00 ± 0.38	0.88 ± 0.29
CXCL12	1.00 ± 0.40	0.80 ± 0.31
CXCL13	1.00 ± 0.48	0.58 ± 0.35

C57BL/6J male mice were treated with or without PRI-724 (0.4 mg/mouse, three times a week) for one week. The expression of the indicated mRNA variants in the isolated IHLs was determined by quantitative real-time RT-PCR. Data are mean ± SD from at least 3 independent experiments. $^{*}P < 0.05$ using a 2-tailed Student's *t*-test.

Supplementary Table 4: Primer sequences used for quantitative real-time RT-PCR

	Forward sequence	Reverse sequence
ATF3	AAGACAGAGTGCCTGCAGAA	GTGCCACCTCTGCTTAGCTC
CCL-2	ATTGGGATCATCTTGCTGGT	CCTGCTGTTCACAGTTGCC
CCL-3	GTGGAATCTTCCGGCTGTAG	ACCATGACACTCTGCAACCA
CCL-4	GAAACAGCAGGAAGTGGGAG	CATGAAGCTCTGCGTGTCTG
CCL-5	CCACTTCTTCTCTGGGTTGG	GTGCCCACGTCAAGGAGTAT
CCL-7	CCTGGGAAGCTGTTATCTTCAA	TGGAGTTGGGGTTTTCATGTC
CCL-8	GAAGGGGGATCTTCAGCTTT	TCTTTGCCTGCTGCTCATAG
CXCL-1	TCTCCGTTACTTGGGGACAC	CCACACTCAAGAATGGTCGC
CXCL-2	TCCAGGTCAGTTAGCCTTGC	CGGTCAAAAAGTTTGCCTTG
CXCL-3	CAGCCACACTCCAGCCTA	CACAACAGCCCCTGTAGC
CXCL-9	CGGACTTCACTCCAACACAG	TAGGGTTCCTCGAACTCCAC
CXCL-10	CCTATGGCCCTCATTCTCAC	CTCATCCTGCTGGGTCTGAG
CXCL-12	TGCACGGCTGAAGAACAACAACAG	TCACACCTCTCACATCTTGAGCCT
CXCL-13	CAGGCCACGGTATTCTGGA	CAGGGGGCGTAACTTGAATC
CD11c	ACACAGTGTGCTCCAGTATGA	GCCCAGGGATATGTTCACAGC
IFN-γ	TGAGCTCATTGAATGCTTGG	ACAGCAAGGCGAAAAAGGAT
CD163	TCCACACGTCCAGAACAGTC	CCTTGGAAACAGAGACAGGC
Mannose receptor	TCTTGGGTCGGATGATTCTG	GCCTGCTCTTCCTCTGACCT
IL-10	GCTCTTACTGACTGGCATGAG	CGCAGCTCTAGGAGCATGTG
